# Learning from Real‐World Data Platforms: Insights into Care and Representation in Research Through ALZ‐NET and New IDEAS

**DOI:** 10.1002/alz.084544

**Published:** 2025-01-09

**Authors:** Charles Windon, Peggye Dilworth‐Anderson, Consuelo H. Wilkins, Maria C. Carrillo, Charles Apgar, Rebecca M. Edelmayer, Constantine Gatsonis, Lucy Hanna, Bruce E Hillner, Andrew March, Sid E. O'Bryant, Michael Rafii, Robert A Rissman, Barry A. Siegel, Heather M Snyder, Rachel A. Whitmer, Gil D. Rabinovici

**Affiliations:** ^1^ Memory and Aging Center, Weill Institute for Neurosciences, University of California, San Francisco, San Francisco, CA USA; ^2^ University of North Carolina, Chapel Hill, Chapel Hill, NC USA; ^3^ Vanderbilt University Medical Center, Nashville, TN USA; ^4^ Alzheimer’s Association, Chicago, IL USA; ^5^ American College of Radiology, Reston, VA USA; ^6^ Brown University, Providence, RI USA; ^7^ Virginia Commonwealth University, Richmond, VA USA; ^8^ University of North Texas Health Science Center, Fort Worth, TX USA; ^9^ Alzheimer’s Therapeutic Research Institute, University of Southern California, San Diego, CA USA; ^10^ Department of Neurosciences, University of California San Diego, La Jolla, CA USA; ^11^ Mallinckrodt Institute of Radiology, Washington University School of Medicine, St. Louis, MO USA; ^12^ University of California, Davis School of Medicine, Sacramento, CA USA; ^13^ Weill Institute for Neurosciences, University of California, San Francisco, San Francisco, CA USA

## Abstract

Real‐World data platforms for Alzheimer’s Disease (AD) offer a unique opportunity to improve health equity through better understanding of health disparities and inclusivity in research, which is critical to translatability of research findings. AD research in the US and globally remains largely inaccessible to many individuals due to individual‐level, study‐level, investigator‐level and larger systemic barriers. ALZ‐NET, a US‐based registry to evaluate longitudinal outcomes of patients being evaluated for or treated with novel FDA‐approved AD therapy, and New IDEAS, an observational US‐based longitudinal study of amyloid PET clinical utility, both offer opportunities for examining care, inclusivity, and disparities.

ALZ‐NET (Alzheimer’s Network for Treatment and Diagnostics) is a national, provider‐enrolled, patient registry collecting longitudinal regulatory grade clinical data. It also features a diagnostic imaging and biospecimen repository. Providers at clinical sites across the US enroll and longitudinally follow patients being considered for Food and Drug Administration (FDA)‐approved AD therapies according to local site practice standards. Clinical practice sites across the US are eligible for ALZ‐NET participation, allowing a greater diversity of participating patients. Registry data is maintained using centralized, secure electronic data capture and management systems that will allow for co‐enrollment of participants into affiliated clinical trials, merging of data with existing databases, and data sharing to facilitate additional research studies.

NEW IDEAS (Imaging Dementia ‐ Evidence for Amyloid Scanning) has enrolled cognitively impaired Medicare beneficiaries at memory clinics across the US since 2020. New IDEAS features a dedicated recruitment and engagement strategy that leverages community partnerships and liaisons in 9 targeted, highly diverse US metro areas to specifically address barriers to participation in traditional research. New IDEAS has identified and developed novel methodology that can inform future studies in successfully increasing representation in AD research on a large scale, with >40% of the current patient population consisting of individuals historically not included in research. New IDEAS also offers insights into rates of amyloid pathology by *in vivo* biomarkers among diverse populations.

Real world data platforms like ALZ‐NET and New IDEAS serve an important role in fostering greater understanding of health disparities and inclusivity in research to improve care.

